# 2,2′-(1,3,5,7-Tetra­oxo-1,2,3,5,6,7-hexa­hydro­pyrrolo[3,4-*f*]isoindole-2,6-di­yl)diacetic acid *N*,*N*-dimethyl­formamide disolvate

**DOI:** 10.1107/S1600536809035107

**Published:** 2009-09-09

**Authors:** Chunhua Ge, Xiangqian Li, Xiangdong Zhang, Yang Zhao, Rui Zhang

**Affiliations:** a College of Chemistry, Liaoning University, Shenyang 110036, People’s Republic of China

## Abstract

The asymmetric unit of the title compound, C_14_H_8_N_2_O_8_·2C_3_H_7_NO or *L*·2DMF (DMF = *N*,*N*-dimethyl­formamide), contains one half of the centrosymmetric mol­ecule *L* and one solvent mol­ecule, which is disordered between two orientations in a 0.555 (4):0.445 (4) ratio. Inter­molecular O—H⋯O hydrogen bonds link one *L* and two DMF mol­ecules into a centrosymmetric hydrogen-bonded cluster. The crystal packing is further stabilized by weak inter­molecular C—H⋯O hydrogen bonds.

## Related literature

For recent developments in the chemistry of naphthalene diimides, see Bhosale *et al.* (2008 ▶). For pyromellitic diimides, see: Gabriel & Iverson (2002 ▶); Ghosh & Ramakrishnan (2005 ▶); Kimizuka *et al.* (1995 ▶). For details of the synthesis, see Barooah *et al.* (2006 ▶).
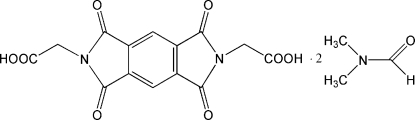


## Experimental

### 

#### Crystal data

C_14_H_8_N_2_O_8_·2C_3_H_7_NO
*M* *_r_* = 478.42
Monoclinic, 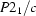

*a* = 7.7470 (15) Å
*b* = 9.3100 (19) Å
*c* = 16.334 (5) Å
β = 104.02 (3)°
*V* = 1143.0 (5) Å^3^
*Z* = 2
Mo *K*α radiation
μ = 0.11 mm^−1^
*T* = 293 K
0.30 × 0.25 × 0.25 mm

#### Data collection

Bruker SMART CCD area-detector diffractometer
Absorption correction: multi-scan (*SADABS*; Sheldrick, 1996 ▶) *T*_min_ = 0.958, *T*_max_ = 0.973
6227 measured reflections
2236 independent reflections
1910 reflections with *I* > 2σ(*I*)
*R*_int_ = 0.030

#### Refinement

*R*[*F* ^2^ > 2σ(*F* ^2^)] = 0.039
*wR*(*F* ^2^) = 0.111
*S* = 1.06
2236 reflections
166 parameters
H-atom parameters constrained
Δρ_max_ = 0.18 e Å^−3^
Δρ_min_ = −0.15 e Å^−3^

### 

Data collection: *SMART* (Bruker, 2001 ▶); cell refinement: *SAINT* (Bruker, 2001 ▶); data reduction: *SAINT*; program(s) used to solve structure: *SHELXS97* (Sheldrick, 2008 ▶); program(s) used to refine structure: *SHELXL97* (Sheldrick, 2008 ▶); molecular graphics: *SHELXTL* (Sheldrick, 2008 ▶); software used to prepare material for publication: *SHELXL97*.

## Supplementary Material

Crystal structure: contains datablocks I, global. DOI: 10.1107/S1600536809035107/cv2607sup1.cif

Structure factors: contains datablocks I. DOI: 10.1107/S1600536809035107/cv2607Isup2.hkl

Additional supplementary materials:  crystallographic information; 3D view; checkCIF report

## Figures and Tables

**Table 1:** Hydrogen-bond geometry (Å, °)

*D*—H⋯*A*	*D*—H	H⋯*A*	*D*⋯*A*	*D*—H⋯*A*
O3—H3*A*⋯O5*A*	0.82	1.72	2.526 (2)	166
O4—H4*B*⋯O5*B*	0.82	1.70	2.496 (2)	162
C2—H2*A*⋯O3^i^	0.97	2.41	3.230 (2)	142
C6—H6⋯O1^ii^	0.93	2.50	3.414 (2)	166
C9—H9*A*⋯O2^iii^	0.96	2.57	3.432 (3)	149

Symmetry codes: (i) 
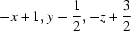
; (ii) 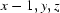; (iii) 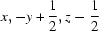.

**Fig. 1.**
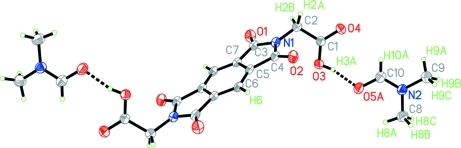
A portion of the crystal structure of the title compound showing the hydrogen-bonded (dashed lines) claster, atomic numbering and 30% probabilty displacement ellipsoids. Unlabelled atoms are related to the labelled ones by symmetry element (1 - x, 2 - y, 1 - z). For the disordered atoms, only major parts are drawn.

## supplementary crystallographic information

### Comment

Interest in the derivatives of diimide such as pyromellitic diimides, naphthalene diimides, and perylene diimides has arisen because of their potential applications for supramolecular and new functional materials (Bhosale *et al.*,  2008). There have been a number of studies investigating the host–guest chemistry about pyromellitic diimide (Gabriel & Iverson, 2002; Ghosh & Ramakrishnan, 2005; Kimizuka *et al.*, 1995). Supramolecular host of *L* and inclusion compounds with aromatic guests have been described (Barooah *et al.*,  2006). In this paper, we report the crystal structure of the title compound, obtained by the recrystallization in DMF-MeOH.

In the molecule *L* (=2,2'-(1,3,5,7-tetraoxo-5,7-dihydropyrrolo[3,4- *f*]isoindole-2,6-diyl)diacetic acid),  two acetic acid groups are placed on upper and lower sides of the rigid conjugate plane (Fig. 1). Intermolecular O—H···O hydrogen bonds (Table 1) link one *L* and two solvent molecules into centrosymmetric hydrogen-bonded cluster. The crystal packing is further stabilized by weak intermolecular C—H···O hydrogen bonds (Table 1).

### Experimental

2,2'-(1,3,5,7-Tetraoxo-5,7-dihydropyrrolo[3,4-*f*]isoindole-2,6-diyl)diacetic acid was synthesized according to the literature (Barooah *et al.*,  2006). *N,N'-*dimethylformamide (DMF) 5 ml was added into a solution of compound mentioned above 0.1 mmol in 20 ml MeOH. The resultant colourless solution was filtered. Crystals suitable for X-ray analysis were formed after three days at room temperature.

### Refinement

All H atoms were placed in calculated positions and included in a riding-model approximation, with C—H = 0.93 - 0.97 Å, O—H = 0.82 Å and *U*_iso_(H)= 1.2-1.5 *U*_eq_ of the parent atom. The solvent molecule is disordered between two orientations with the occupancies refined to 0.555 (4) and 0.445 (4), respectively. The hydroxy H atom is also disordered between two positions - H3A and H4B - with the same occupancies, respectively.

### Crystal data

**Table:**

C_14_H_8_N_2_O_8_·2C_3_H_7_NO	*F*(000) = 500
*M**_r_* = 478.42	*D*_x_ = 1.390 Mg m^−^^3^
Monoclinic, *P*2_1_/*c*	Mo *K*α radiation, λ = 0.71073 Å
Hall symbol: -P 2ybc	Cell parameters from 541 reflections
*a* = 7.7470 (15) Å	θ = 2.5–22.7°
*b* = 9.3100 (19) Å	µ = 0.11 mm^−^^1^
*c* = 16.334 (5) Å	*T* = 293 K
β = 104.02 (3)°	Block, colourless
*V* = 1143.0 (5) Å^3^	0.30 × 0.25 × 0.25 mm
*Z* = 2	

### Data collection

**Table:**

Bruker SMART CCD area-detector diffractometer	2236 independent reflections
Radiation source: fine-focus sealed tube	1910 reflections with *I* > 2σ(*I*)
graphite	*R*_int_ = 0.030
φ and ω scans	θ_max_ = 26.0°, θ_min_ = 2.5°
Absorption correction: multi-scan (*SADABS*; Sheldrick, 1996)	*h* = −9→9
*T*_min_ = 0.958, *T*_max_ = 0.973	*k* = −11→10
6227 measured reflections	*l* = −20→18

### Refinement

**Table:**

Refinement on *F*^2^	Primary atom site location: structure-invariant direct methods
Least-squares matrix: full	Secondary atom site location: difference Fourier map
*R*[*F*^2^ > 2σ(*F*^2^)] = 0.039	Hydrogen site location: inferred from neighbouring sites
*wR*(*F*^2^) = 0.111	H-atom parameters constrained
*S* = 1.06	*w* = 1/[σ^2^(*F*_o_^2^) + (0.0528*P*)^2^ + 0.2565*P*] where *P* = (*F*_o_^2^ + 2*F*_c_^2^)/3
2236 reflections	(Δ/σ)_max_ < 0.001
166 parameters	Δρ_max_ = 0.18 e Å^−^^3^
0 restraints	Δρ_min_ = −0.15 e Å^−^^3^

### Special details

**Table:**

Geometry. All e.s.d.'s (except the e.s.d. in the dihedral angle between two l.s. planes) are estimated using the full covariance matrix. The cell e.s.d.'s are taken into account individually in the estimation of e.s.d.'s in distances, angles and torsion angles; correlations between e.s.d.'s in cell parameters are only used when they are defined by crystal symmetry. An approximate (isotropic) treatment of cell e.s.d.'s is used for estimating e.s.d.'s involving l.s. planes.
Refinement. Refinement of *F*^2^ against ALL reflections. The weighted *R*-factor *wR* and goodness of fit *S* are based on *F*^2^, conventional *R*-factors *R* are based on *F*, with *F* set to zero for negative *F*^2^. The threshold expression of *F*^2^ > σ(*F*^2^) is used only for calculating *R*-factors(gt) *etc*. and is not relevant to the choice of reflections for refinement. *R*-factors based on *F*^2^ are statistically about twice as large as those based on *F*, and *R*- factors based on ALL data will be even larger.

### Fractional atomic coordinates and isotropic or equivalent isotropic displacement parameters (Å^2^)

**Table:**

	*x*	*y*	*z*	*U*_iso_*/*U*_eq_	Occ. (<1)
O3	0.46550 (18)	0.49571 (11)	0.66856 (7)	0.0615 (3)	
H3A	0.4139	0.5238	0.6214	0.061*	0.555 (4)
O4	0.48981 (19)	0.29416 (13)	0.59801 (7)	0.0689 (4)	
H4B	0.4357	0.3423	0.5579	0.069*	0.445 (4)
O1	0.86755 (14)	0.43154 (15)	0.88375 (8)	0.0638 (4)	
O2	0.27767 (14)	0.31116 (13)	0.79491 (7)	0.0573 (3)	
N1	0.58016 (15)	0.35733 (14)	0.82137 (7)	0.0451 (3)	
C1	0.51315 (19)	0.36686 (16)	0.66427 (9)	0.0457 (3)	
C2	0.6117 (2)	0.29280 (17)	0.74458 (10)	0.0504 (4)	
H2A	0.5759	0.1928	0.7422	0.060*	
H2B	0.7383	0.2956	0.7476	0.060*	
C3	0.71166 (19)	0.42013 (16)	0.88520 (9)	0.0447 (3)	
C4	0.41265 (18)	0.35918 (15)	0.84052 (9)	0.0429 (3)	
C5	0.43776 (17)	0.43160 (14)	0.92473 (8)	0.0388 (3)	
C6	0.31300 (17)	0.46298 (15)	0.97165 (9)	0.0410 (3)	
H6	0.1933	0.4390	0.9531	0.049*	
C7	0.61865 (17)	0.46738 (15)	0.95191 (8)	0.0390 (3)	
N2	0.09136 (18)	0.56664 (15)	0.40440 (9)	0.0545 (4)	
C10	0.2173 (2)	0.5240 (2)	0.46976 (11)	0.0592 (4)	
H10A	0.2662	0.4330	0.4691	0.071*	0.555 (4)
H10B	0.2583	0.5849	0.5155	0.071*	0.445 (4)
C9	0.0213 (3)	0.4691 (2)	0.33408 (13)	0.0726 (5)	
H9A	0.0839	0.3793	0.3439	0.109*	
H9B	0.0370	0.5110	0.2827	0.109*	
H9C	−0.1031	0.4529	0.3295	0.109*	
C8	0.0168 (3)	0.7115 (2)	0.40126 (13)	0.0707 (5)	
H8A	0.0662	0.7600	0.4536	0.106*	
H8B	−0.1101	0.7056	0.3925	0.106*	
H8C	0.0454	0.7640	0.3557	0.106*	
O5A	0.2757 (3)	0.6081 (2)	0.53640 (13)	0.0644 (8)	0.555 (4)
O5B	0.2835 (4)	0.3945 (3)	0.46915 (17)	0.0705 (11)	0.445 (4)

### Atomic displacement parameters (Å^2^)

**Table:**

	*U*^11^	*U*^22^	*U*^33^	*U*^12^	*U*^13^	*U*^23^
O3	0.0914 (9)	0.0416 (6)	0.0451 (6)	0.0103 (6)	0.0044 (6)	−0.0013 (5)
O4	0.0965 (10)	0.0517 (7)	0.0497 (7)	0.0094 (6)	0.0004 (6)	−0.0124 (5)
O1	0.0363 (6)	0.0933 (9)	0.0612 (7)	−0.0030 (6)	0.0105 (5)	−0.0050 (6)
O2	0.0464 (6)	0.0670 (7)	0.0513 (6)	−0.0117 (5)	−0.0021 (5)	−0.0040 (5)
N1	0.0416 (6)	0.0520 (7)	0.0393 (6)	−0.0001 (5)	0.0049 (5)	0.0040 (5)
C1	0.0469 (8)	0.0430 (8)	0.0452 (8)	−0.0043 (6)	0.0074 (6)	0.0013 (6)
C2	0.0553 (9)	0.0465 (8)	0.0478 (8)	0.0061 (7)	0.0096 (7)	0.0031 (6)
C3	0.0373 (7)	0.0499 (8)	0.0432 (7)	0.0010 (6)	0.0025 (6)	0.0091 (6)
C4	0.0407 (7)	0.0417 (7)	0.0413 (7)	−0.0020 (6)	0.0006 (6)	0.0092 (6)
C5	0.0345 (7)	0.0380 (7)	0.0391 (7)	−0.0020 (5)	−0.0007 (5)	0.0102 (5)
C6	0.0291 (6)	0.0451 (7)	0.0439 (7)	−0.0036 (5)	−0.0010 (5)	0.0088 (6)
C7	0.0321 (7)	0.0408 (7)	0.0409 (7)	0.0004 (5)	0.0027 (5)	0.0108 (6)
N2	0.0486 (7)	0.0539 (8)	0.0562 (8)	0.0005 (6)	0.0033 (6)	0.0000 (6)
C10	0.0557 (10)	0.0610 (10)	0.0573 (10)	0.0013 (8)	0.0069 (8)	0.0063 (8)
C9	0.0696 (12)	0.0693 (12)	0.0691 (12)	0.0050 (10)	−0.0019 (9)	−0.0119 (9)
C8	0.0690 (12)	0.0566 (10)	0.0766 (12)	0.0060 (9)	−0.0016 (9)	−0.0009 (9)
O5A	0.0737 (15)	0.0581 (13)	0.0527 (13)	0.0055 (11)	−0.0016 (10)	0.0063 (10)
O5B	0.081 (2)	0.069 (2)	0.0497 (16)	0.0120 (15)	−0.0070 (13)	0.0009 (13)

### Geometric parameters (Å, °)

**Table:**

O3—C1	1.2621 (18)	C6—C7^i^	1.393 (2)
O3—H3A	0.8200	C6—H6	0.9300
O4—C1	1.2519 (18)	C7—C6^i^	1.393 (2)
O4—H4B	0.8200	N2—C10	1.321 (2)
O1—C3	1.2182 (18)	N2—C9	1.462 (2)
O2—C4	1.2128 (17)	N2—C8	1.463 (2)
N1—C3	1.3971 (19)	C10—O5B	1.311 (3)
N1—C4	1.4067 (19)	C10—O5A	1.328 (3)
N1—C2	1.464 (2)	C10—H10A	0.9300
C1—C2	1.515 (2)	C10—H10B	0.9300
C2—H2A	0.9700	C9—H9A	0.9600
C2—H2B	0.9700	C9—H9B	0.9600
C3—C7	1.510 (2)	C9—H9C	0.9600
C4—C5	1.502 (2)	C8—H8A	0.9600
C5—C6	1.402 (2)	C8—H8B	0.9600
C5—C7	1.4037 (19)	C8—H8C	0.9600
			
C1—O3—H3A	109.5	C6^i^—C7—C5	121.85 (13)
C1—O4—H4B	109.5	C6^i^—C7—C3	129.67 (12)
C3—N1—C4	111.97 (12)	C5—C7—C3	108.48 (12)
C3—N1—C2	124.62 (13)	C10—N2—C9	120.63 (15)
C4—N1—C2	123.38 (13)	C8—N2—C10	120.88 (15)
O4—C1—O3	125.38 (14)	C9—N2—C8	118.48 (14)
O4—C1—C2	116.07 (13)	O5B—C10—N2	118.93 (19)
O3—C1—C2	118.53 (13)	O5B—C10—O5A	119.5 (2)
N1—C2—C1	113.67 (12)	N2—C10—O5A	121.58 (18)
N1—C2—H2A	108.8	N2—C10—H10A	119.2
C1—C2—H2A	108.8	O5A—C10—H10A	119.2
N1—C2—H2B	108.8	O5B—C10—H10B	120.5
C1—C2—H2B	108.8	N2—C10—H10B	120.5
H2A—C2—H2B	107.7	H10A—C10—H10B	120.2
O1—C3—N1	124.69 (14)	N2—C9—H9A	109.5
O1—C3—C7	129.57 (14)	N2—C9—H9B	109.5
N1—C3—C7	105.74 (12)	H9A—C9—H9B	109.5
O2—C4—N1	124.30 (14)	N2—C9—H9C	109.5
O2—C4—C5	128.99 (14)	H9A—C9—H9C	109.5
N1—C4—C5	106.70 (11)	H9B—C9—H9C	109.5
C6—C5—C7	123.04 (13)	N2—C8—H8A	109.5
C6—C5—C4	129.85 (12)	N2—C8—H8B	109.5
C7—C5—C4	107.11 (13)	H8A—C8—H8B	109.5
C7^i^—C6—C5	115.11 (12)	N2—C8—H8C	109.5
C7^i^—C6—H6	122.4	H8A—C8—H8C	109.5
C5—C6—H6	122.4	H8B—C8—H8C	109.5
			
C3—N1—C2—C1	−117.78 (16)	N1—C4—C5—C7	−0.77 (14)
C4—N1—C2—C1	64.21 (19)	C7—C5—C6—C7^i^	−0.1 (2)
O4—C1—C2—N1	−158.82 (14)	C4—C5—C6—C7^i^	−179.65 (13)
O3—C1—C2—N1	22.9 (2)	C6—C5—C7—C6^i^	0.1 (2)
C4—N1—C3—O1	179.90 (15)	C4—C5—C7—C6^i^	179.75 (12)
C2—N1—C3—O1	1.7 (2)	C6—C5—C7—C3	−178.94 (12)
C4—N1—C3—C7	−0.18 (16)	C4—C5—C7—C3	0.67 (14)
C2—N1—C3—C7	−178.38 (12)	O1—C3—C7—C6^i^	0.6 (3)
C3—N1—C4—O2	179.89 (14)	N1—C3—C7—C6^i^	−179.32 (13)
C2—N1—C4—O2	−1.9 (2)	O1—C3—C7—C5	179.59 (15)
C3—N1—C4—C5	0.58 (15)	N1—C3—C7—C5	−0.33 (15)
C2—N1—C4—C5	178.81 (12)	C9—N2—C10—O5B	2.6 (3)
O2—C4—C5—C6	−0.5 (2)	C8—N2—C8—O5B	−178.4 (2)
N1—C4—C5—C6	178.80 (13)	C9—N2—C10—O5A	−176.1 (2)
O2—C4—C5—C7	179.96 (15)	C8—N2—C8—O5A	2.9 (3)

Symmetry codes: (i) −*x*+1, −*y*+1, −*z*+2.

### Hydrogen-bond geometry (Å, °)

**Table:**

*D*—H···*A*	*D*—H	H···*A*	*D*···*A*	*D*—H···*A*
O3—H3A···O5A	0.82	1.72	2.526 (2)	166
O4—H4B···O5B	0.82	1.70	2.496 (2)	162
C2—H2A···O3^ii^	0.97	2.41	3.230 (2)	142
C6—H6···O1^iii^	0.93	2.50	3.414 (2)	166
C9—H9A···O2^iv^	0.96	2.57	3.432 (3)	149

Symmetry codes:  (ii) −*x*+1, *y*−1/2, −*z*+3/2; (iii) *x*−1, *y*, *z*; (iv) *x*, −*y*+1/2, *z*−1/2.
